# Subtraction of Temporally Sequential Digital Mammograms: Prediction and Localization of Near-Term Breast Cancer Occurrence

**DOI:** 10.1007/s10278-025-01456-z

**Published:** 2025-03-07

**Authors:** Kosmia Loizidou, Galateia Skouroumouni, Gabriella Savvidou, Anastasia Constantinidou, Eleni Orphanidou Vlachou, Anneza Yiallourou, Costas Pitris, Christos Nikolaou

**Affiliations:** 1 KIOS Research and Innovation Center of Excellence, Department of Electrical and Computer Engineering, University of Cyprus, Nicosia, Cyprus; 2 Ygia Polyclinic Limassol, Limassol, Cyprus; 3 Medical School, University of Cyprus, Bank of Cyprus Oncology Centre, Nicosia, Cyprus; 4 EIMC Clinic Strovolos, Nicosia, Cyprus; 5 Medical School, University of Cyprus, Breast Unit, Nicosia General Hospital, State Health Services Organization, Nicosia, Cyprus; 6 Limassol General Hospital, Limassol, Cyprus

**Keywords:** Breast cancer, Mammography, Machine learning, Prediction, Medical image analysis

## Abstract

The objective is to predict a possible near-term occurrence of a breast mass after two consecutive screening rounds with normal mammograms. For the purposes of this study, conducted between 2020 and 2024, three consecutive rounds of mammograms were collected from 75 women, 46 to 79 years old. Successive screenings had an average interval of $\sim$2 years. In each case, two mammographic views of each breast were collected, resulting in a dataset with a total of 450 images (3 × 2 × 75). The most recent mammogram was considered the “future” screening round and provided the location of a biopsy-confirmed malignant mass, serving as the ground truth for the training. The two normal previous mammograms (“prior” and “current”) were processed and a new subtracted image was created for the prediction. Region segmentation and post-processing were, then, applied, along with image feature extraction and selection. The selected features were incorporated into several classifiers and by applying leave-one-patient-out and k-fold cross-validation per patient, the regions of interest were characterized as benign or possible future malignancy. Study participants included 75 women (mean age, 62.5 ± 7.2; median age, 62 years). Feature selection from benign and possible future malignancy areas revealed that 14 features provided the best classification. The most accurate classification performance was achieved using ensemble voting, with 98.8% accuracy, 93.6% sensitivity, 98.8% specificity, and 0.96 AUC. Given the success of this algorithm, its clinical application could enable earlier diagnosis and improve prognosis for patients identified as at risk.

**Supplementary Information:**

The online version contains supplementary material available at 10.1007/s10278-025-01456-z.

## Introduction

The World Health Organization considers breast cancer (BC) to be a significant global health concern [1]. Mammography, combined with physical examination, is an effective screening modality for the management of BC. The radiological classification of breast abnormalities relies on various parameters including shape, texture, and temporal evaluation of the lesions [2].

However, despite advancements in technology, diagnosing BC remains challenging due to the elusive radiologic nature of breast lesions and the limited imaging contrast [3]. Currently, the assessment of mammograms requires agreement between two expert radiologists, with a third opinion to resolve discrepancies. These multiple readings contribute to increased cost and resource burden. They also exacerbate inefficiencies in the screening process, leading to substantial population under screening.

Over the past three decades, computer-aided diagnosis (CAD) systems have been in development for the detection and classification of BC using the most recent mammograms [4]. However, using only the most recent mammograms does not allow comparison of the recent and prior images of the same patient. Such comparisons are routinely performed by radiologists in order to more effectively identify any abnormalities, which have developed between screenings. Temporal subtraction, developed by this group, was successfully applied for the detection and classification of breast abnormalities using sequential mammograms [5–7].

Accurately identifying breast masses at an early stage, without the need of recall, remains a significant challenge even for well-trained radiologists [3]. It is estimated that between 10% to 30% of BC cases are missed, resulting in a false negative rate of up to 50% [8]. High breast density poses a further diagnostic hurdle as the visibility of abnormal lesions is diminished, thereby reducing mammography sensitivity by approximately 30% [9]. Moreover, retrospective analysis indicates that approximately half of prior mammograms, previously assessed as normal, exhibit signs of abnormality [10]. This led to retrospective studies of prior mammograms, initially classified as normal but later diagnosed with BC, to estimate the risk of future malignancy and understand the timeline of suspicious abnormality evolution [11]. Support vector machines (SVMs) were exploited for BC risk prediction [12–14] with the highest per patient performance, 0.74 AUC, achieved by Sun et al., [12]. Tan et al., [13], collected a private dataset with sequential images from 335 patients. Using SVM and leave-one-out cross-validation (CV) per patient, they achieved a 0.73 AUC [13]. Deep learning (DL) has also been applied for the prediction of BC risk, with convolutional neural networks (CNNs) being the most popular classifiers utilized for this task [4, 15–17]. Yala et al. [16] created a robust mammography-based model for the analysis of BC risk, using ResNet18. A massive dataset was collected, including consecutive screening mammograms from 125,991 patients. The 2-year risk prediction AUC, using a test set of 7005 patients, was 0.80 AUC, which was higher compared to other CNN-based studies.

Recently, CAD systems were also being investigated for the prediction or early diagnosis of BC, to enhance patient prognosis and outcomes [14, 16–18]. However, the reported performance of these studies is not perfect. Most CAD systems and AI-based models currently in use in clinical practice reach an AUC in the range of 0.65–0.8, which is not sufficient for reliable standalone clinical use. Thus, these systems are primarily employed as second readers, providing decision support to the radiologists. This level of performance highlights the need for further innovation in this field.

**Fig. 1:**
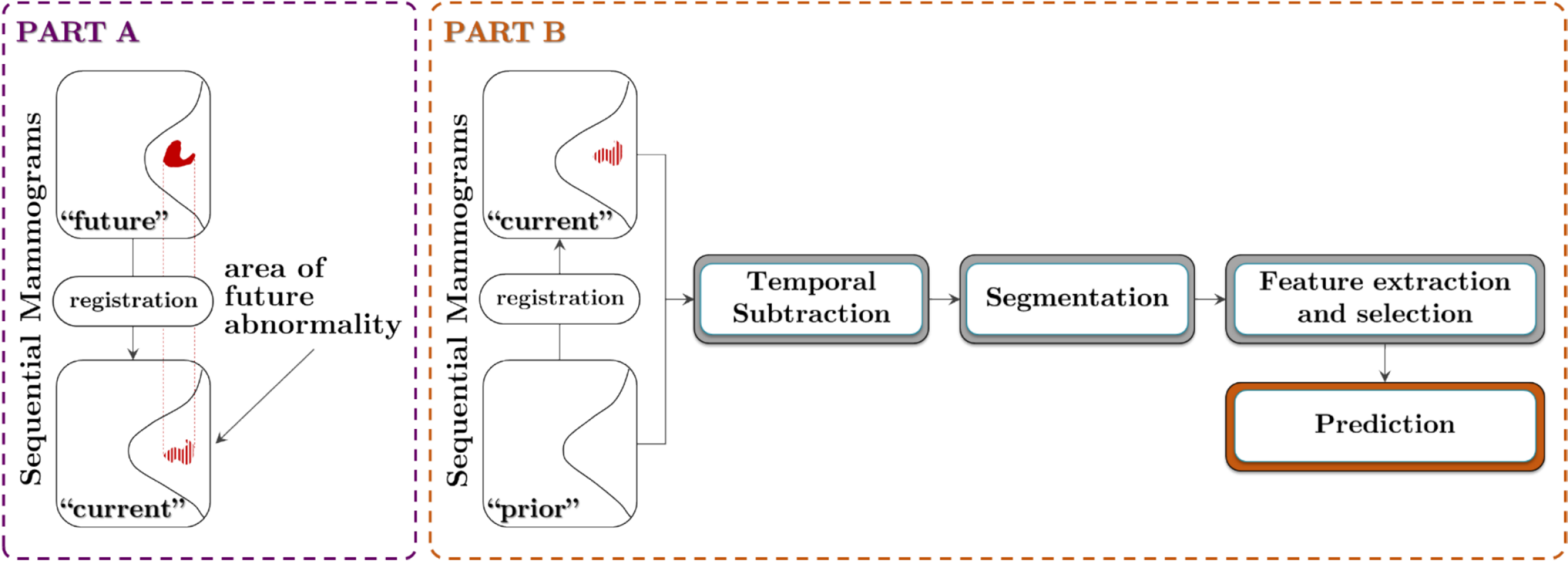
Diagram of the proposed methodology for predicting near-term breast cancer occurrence using subtraction of temporally sequential digital mammograms. **PART A**: Identification of the “future” mass location in the “current” mammogram to establish the ground truth. **PART B**: Algorithm predicting which segmented regions of interest (ROIs) are likely to develop into a mass in the future

This study assessed the effectiveness of temporal subtraction, combined with advanced feature selection and machine learning techniques, for the prediction of near-term BC occurrence. The proposed methodology is illustra**.** ted in Fig. 1.

## Materials and Methods

### Study Design and Data Collection

Twelve patients, out of the 75, have been previously reported in a conference paper [18] that provided an early demonstration of temporal subtraction for predicting BC, aiming to test the feasibility of the approach for further investigation. The current study includes a larger cohort of 75 patients and implements a more rigorous validation of the results, significantly expanding the analysis.

**Fig. 2:**
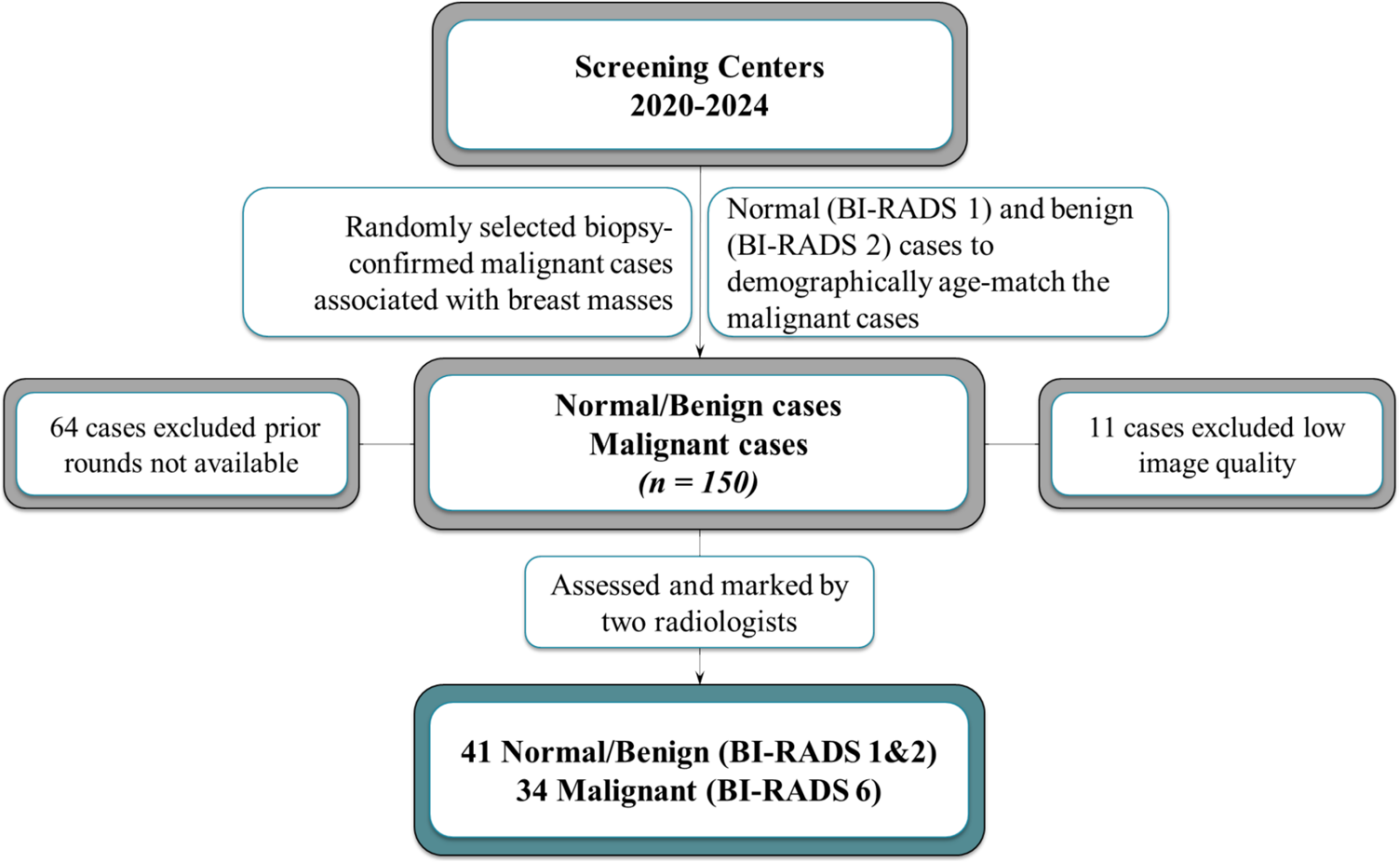
Study flowchart, illustrating the inclusion and exclusion criteria, and the recruitment process

This study, conducted between 2020 and 2024, included women 46 to 79 years of age (mean ± standard deviation, 62.5 ± 7.2). Three consecutive rounds of digital mammograms were collected per participant. In total, 150 cases were collected from various local hospitals (Fig. 2). Altogether, 75 cases were excluded since 64 did not have any prior rounds available and 11 were of low image quality (scanned instead of digital). Ethical considerations were addressed, and the study received approval from the Institutional Review Board. Informed consent was collected during the most recent screening round.

**Fig. 3:**
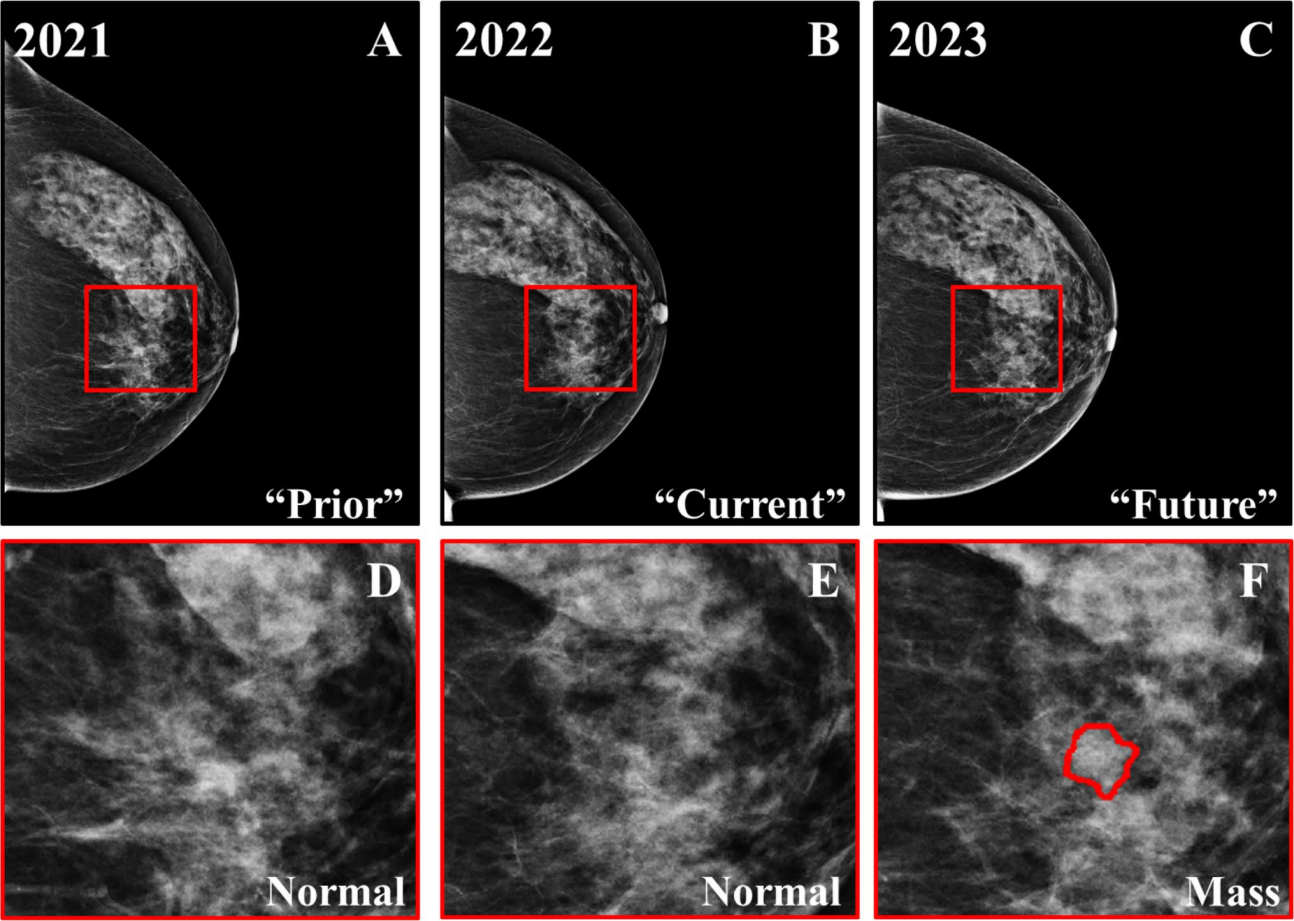
Three sequential screening rounds of a 69-year-old woman (BI-RADS breast density category *c*). **A** “Prior” mammogram from two screening rounds ago. **B **“Current” mammogram from one screening ago. **C** Most recent mammogram (“future”) with a biopsy-confirmed malignant mass. **D–F** Zoomed regions marked by the red squares in **A–C**. **F** The zoomed region marked by the red square in **C** with precise marking of the biopsy-confirmed malignant mass location, as annotated by two expert radiologists

For every participant, two mammographic views of the breast (cranio-caudal (CC) and medio-lateral oblique (MLO)) from three sequential screening rounds were included, resulting in a total of 450 images (3 rounds × 2 views × 75 cases). Two clinicians (C.N., radiologist with 27 years of experience, and A.Y., consultant breast surgeon with 10 years of experience) identified the eligible patients. Two radiologists (G.Sk. with 6 years of experience and E.O.V. with 5 years of experience) outlined the border of each mass for both breast imaging reporting and data system (BI-RADS) benign and suspicious (Fig. 3). Subsequently, suspicious cases were biopsied, followed by histopathologic analysis, confirming their malignant nature. The biopsy confirmations were performed by each hospital’s pathology department, as per standard of care. The information was collected by a clinician and a researcher (A.C., academic medical oncologist with over 10 years of experience and G.Sa., postgraduate researcher at the medical school with 5 years of experience). Table 1 pro vides a summary of the study population.

**Table 1:** Characteristics of the population selected for the study

Variable	Population		
	Normal	Malignant	Total
	*(n* = *41)*	*(n* = *34)*	*(n* = *75)*
**Patient age**			
* Mean* ± *STD*	61.8 ± 8.1	63.1 ± 5.7	62.5 ± 7.2
* Median*	62	62.5	62
* Range*	46 – 79	51 – 77	46 – 79
* Interquartile range*	56 – 69	59.7 – 68	58 – 68
**BI-RADS breast density**			
* a*	9	4	13
* b*	14	15	29
* c*	15	14	29
* d*	3	1	4

*STD* standard deviation

Out of 75 cases, 34 had biopsy-confirmed malignant masses in the most recent mammogram, with two normal prior mammograms. The remaining 41 cases came from participants without any suspicious findings in the most-recent mammogram (BI-RADS 1 & 2), also with two normal prior mammograms. The normal cases were selected to form a group matched to the population of patients with malignant findings. For the purposes of this study, the most recent mammogram was considered the “future” screening round. It provided the location of the biopsy-confirmed malignant mass, which served as the ground truth for the training. The two normal previous mammograms were considered the “prior” and the “current” mammographic screenings. The dataset generated during the current study will be publicly available through Zenodo. Currently, the datasets used and/or analyzed during the current study are available from the corresponding author on reasonable request.

### Localization of the “Future” Mass Location on the “Current” Mammograms

**Fig. 4:**
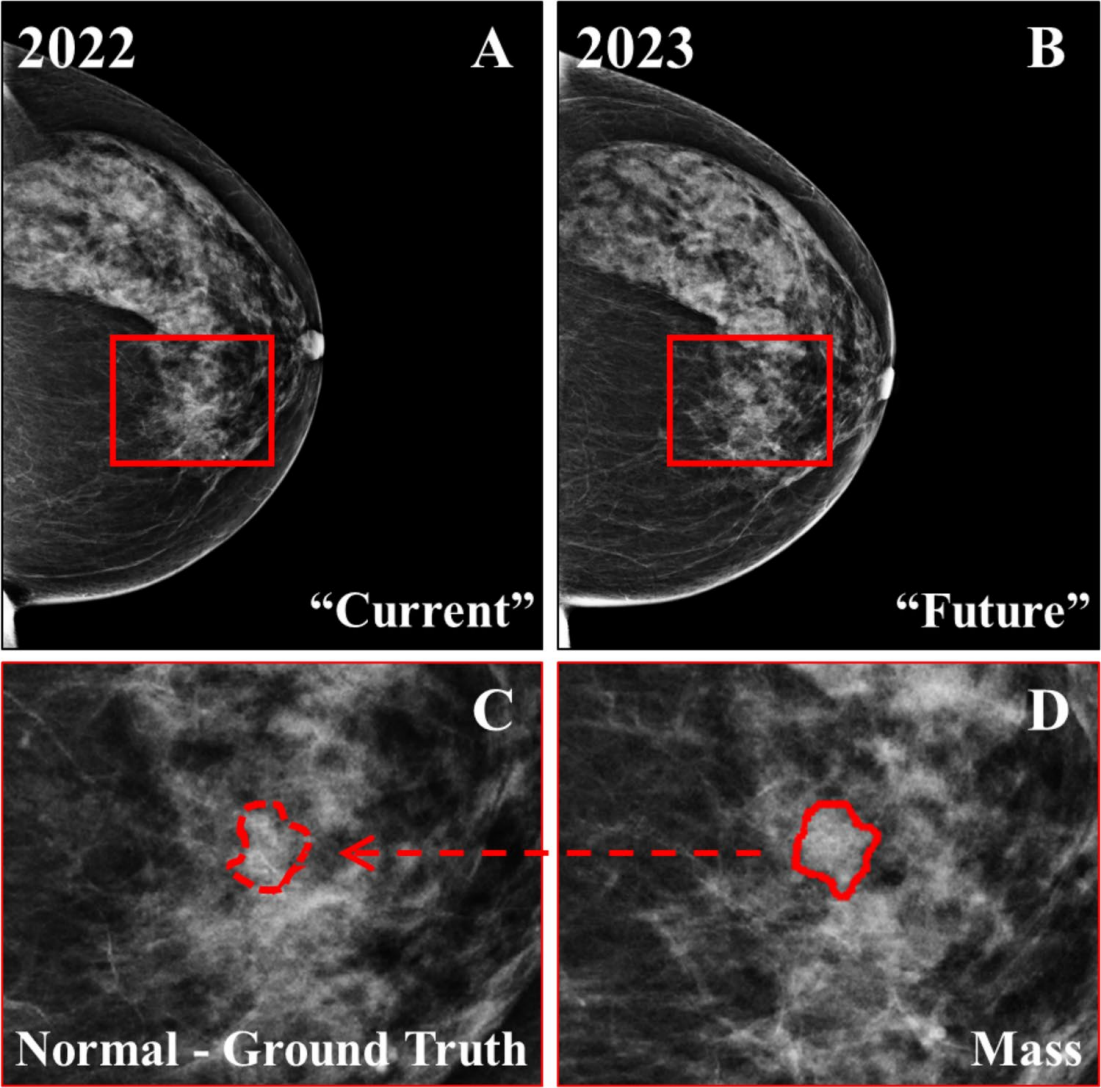
**A** “Current” mammogram of a 69-year-old woman (BI-RADS breast density category *c*) showing the location where the mass would appear during the following screening round, found using image registration between the “future” and “current” images. **B** “Future” mammogram with a biopsy-confirmed malignant mass. **C–D** Zoomed regions marked by the red squares in **A–B**. No radiological signs of malignancy are visible in **B** or **D**

A crucial step in the training of the algorithm was to identify the locations corresponding to the “future” masses, in the 34 cases associated with malignant findings, in the “current” images. The process started with the pre-processing using normalization, contrast limited adaptive histogram equalization (CLAHE), gamma correction, and border removal (Fig. S1). Demons registration was applied to register the “future” to the “current” image and, thus, pinpoint the location corresponding to the biopsy-confirmed malignant mass in the “current” image [19]. The area where the mass would appear in the next screening round was marked on the “current” mammogram, even though no abnormality was visible (Fig. 4). These marked areas in the “current” image were then used as the ground truth for the training of the algorithm and the “future” images were not further considered in the analysis.

### Processing and Analysis of the “Prior” and “Current” Mammograms

**Fig. 5:**
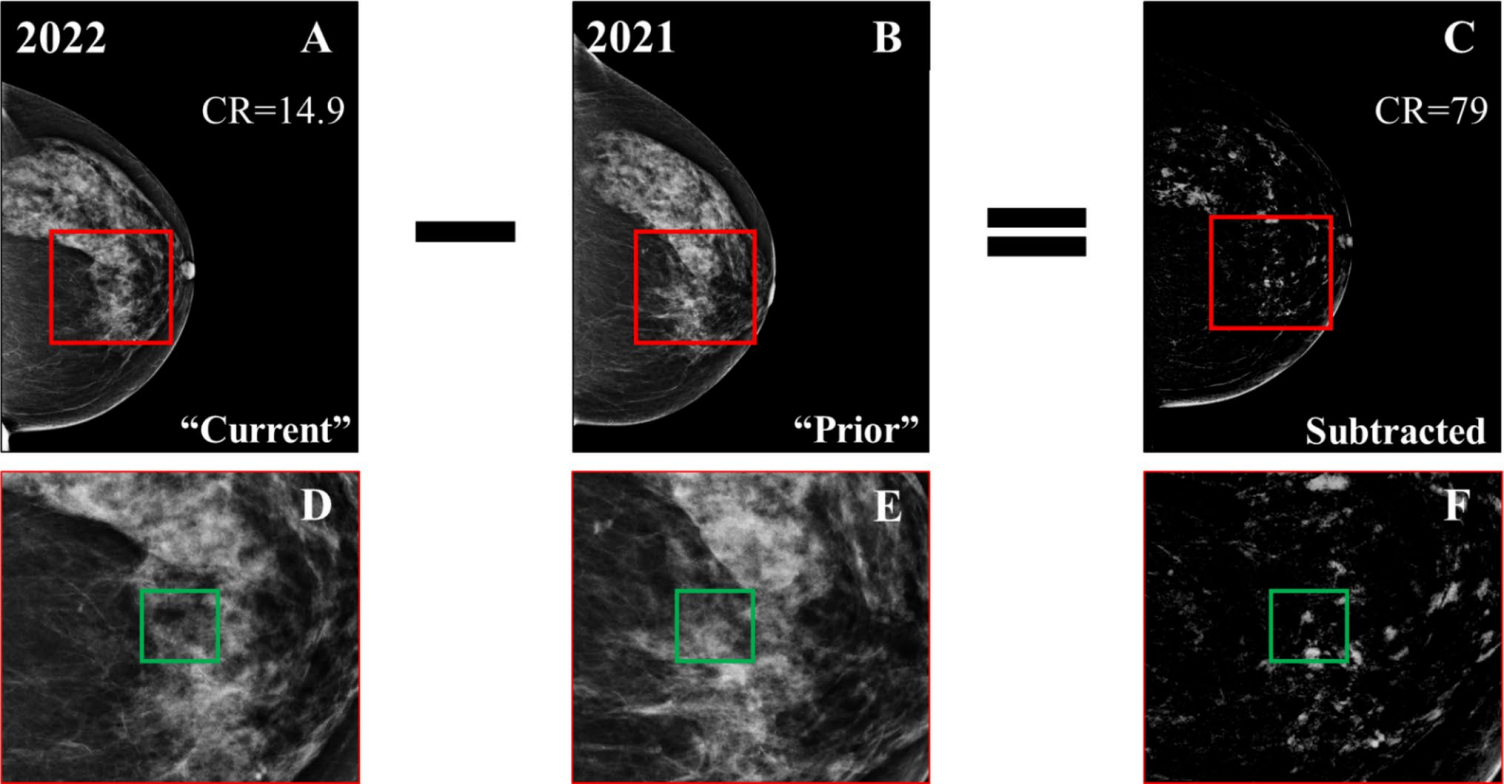
Example of temporal subtraction in a 69-year-old woman (BI-RADS breast density category *c*). **A** “Current” mammogram. **B** “Prior” mammogram. Neither **A** or **B** exhibit a visible malignancy. **C** The result of subtracting the registered version of **B** from **A**. **D–F** Zoomed regions marked by the red squares in **A–C**, where the green squares show the location of a biopsy-confirmed malignant mass that emerged in the next screening (“future”) round. The contrast ratio (CR) has increased five times after subtraction

After establishing the ground truth, by identifying the mass locations for the 34 malignant cases, the focus shifted to the “prior” and “current” mammograms of all cases. The objective was to subtract those two images effectively (Fig. 5), identify changes, and segment all new regions of interest (ROIs).

Key steps included pre-processing (normalization, CLAHE, gamma correction, and border removal, as before) to enhance the image quality, Demons registration to align the “prior” mammogram with the “current” one [19], and subtraction of the “prior” registered image from the “current,” to highlight new changes. Additional processing was applied to the newly created subtracted images, using unsharp-mask filtering, to enhance the high spatial frequencies [20].

Segmentation isolated the ROIs for further analysis applying the following steps: (1) thresholding, (2) application of morphological operations, and (3) removal of periphery pixels. The proccess started with thresholding, which converted the image into binary, to eliminate low-intensity normal areas. The threshold value was selected using histogram analysis and optimization of the global classification rate, during training. Next, morphological operations were applied to the binary image. Erosion was used to remove isolated pixels resulting from registration misalignment or thresholding artifacts, while closing was used to merge neighboring pixels. Erosion was performed with a 2-pixel radius, smaller than the average mass size, to avoid merging of adjacent ROIs. Finally, closing was performed with a 10-pixel radius to identify the constituents of each mass. The radius size was chosen after comparing with the ground truth. High-intensity regions in the periphery of the breast that, most likely, correspond to the skin of the breast were removed, since masses cannot form in that region. The whole breast region was binarized and its border was identified. All high-intensity regions along the border were removed. The algorithm considered the remaining regions as the ROIs.

For each ROI identified, 98 diverse features were collected including both epidemiological and image characteristics (shape-based, intensity-based, first-order statistics (FOS), and gray level co-occurrence matrix (GLCM) features). Each GLCM feature was derived at 0, 45, 90, and 135 degrees, with the mean and standard deviation (STD) also calculated, i.e., 24 values for each offset *D* (*D*_*1*_ = *5*,* D*_*2*_ = *15*, and* D*_*3*_ = *25* pixels). Eight feature selection techniques were applied, to identify the most predictive features for distinguishing normal from “future” malignant ROIs. A majority rule approach was employed to merge the multiple rankings obtained (Table 2).

**Table 2:** Ranking of the features for the classification round using different feature selection techniques. The final selection, using the majority rule, is shown in bold

*t*-test	MRMR	FI-ET	FI-RF	FI-XGB	SelectKBest	SFS	SBS
**Area**	**Minor Axis Length**	**Area**	**Area**	**Area**	**Area**	Orientation	**Area**
Major Axis Length	**Euler Number**	**Minor Axis Length**	**Minor Axis Length**	**Minor Axis Length**	Major Axis Length	**Convex Area**	**Filled Area**
**Minor Axis Length**	Solidity	**Convex Area**	**Euler Number**	**Euler Number**	**Minor Axis Length**	**Euler Number**	**Euler Number**
**Convex Area**	Contrast STD D1	**Filled Area**	**Equivalent Diameter**	**Perimeter**	**Convex Area**	Solidity	**Perimeter**
**Filled Area**	**Energy STD D1**	**Euler Number**	Solidity	Contrast 45 D1	**Filled Area**	Extent	**Shape Ratio**
**Equivalent Diameter**	Homogeneity STD D1	**Equivalent Diameter**	**Perimeter**	Correlation STD D1	**Equivalent Diameter**	Mean Intensity	
**Perimeter**	Contrast STD D2	**Perimeter**	Max Intensity	**Energy STD D1**	**Perimeter**	Min Intensity	
Energy 45 D2	Energy STD D2	Mean Intensity	STD	Homogeneity STD D1	Energy 45 D2	Contrast 45 D2	
Energy 135 D2	Homogeneity 45 D2	Max Intensity	**Energy STD D1**	Contrast 135 D2	Energy 135 D2	Correlation 90 D2	
Energy Mean D2	Homogeneity STD D2	STD	Homogeneity STD D1	Correlation 0 D2	Energy Mean D2	Energy 45 D2	
Energy 0 D3	Contrast 90 D3	Variance	Energy STD D2	Homogeneity 45 D2	Energy 0 D3	Contrast 45 D3	
**Energy 45 D3**	Contrast STD D3	**Energy STD D1**	Homogeneity STD D2	Homogeneity 135 D2	**Energy 45 D3**	Contrast 135 D3	
Energy 90 D3	Correlation 45 D3	Energy STD D2	**Energy 45 D3**	Homogeneity STD D2	Energy 90 D3	**Circularity**	
**Energy 135 D3**	Correlation 135 D3	**Energy 45 D3**	**Energy 135 D3**	Correlation 135 D3	**Energy 135 D3**	**Compactness**	
Energy Mean D3	Correlation Mean D3	**Energy 135 D3**	Energy STD D3	Energy 90 D3	Energy Mean D3		
**Homogeneity 135 D3**	**Energy 45 D3**	Energy STD D3	**Homogeneity 135 D3**	Homogeneity 0 D3	**Homogeneity 135 D3**		
Homogeneity Mean D3	Energy STD D3	Homogeneity 45 D3	Homogeneity STD D3	**Homogeneity 135 D3**	Homogeneity Mean D3		
**Circularity**	Homogeneity 0 D3	**Circularity**	**Circularity**	Homogeneity Mean D3	**Circularity**		
**Compactness**	**Homogeneity 135 D3**	**Compactness**	**Shape Ratio**	Homogeneity STD D3	**Compactness**		
**Shape Ratio**	Homogeneity STD D3	Smoothness	**Smoothness**	Breast Density	**Shape Ratio**		

*MRMR:* maximum relevance-minimum redundancy; *FI-ET:* feature importance using extra trees; *FI-RF:* feature importance using random forest; *FI-XGB:* feature importance using

XGBoost; *SFS:* sequential forward selection; *SBS:* sequential backward selection; *STD:* standard deviation

Ten classifiers were evaluated to predict “future” malignancies. Leave-one-patient-out (LOPO) and k-fold (*k* = 5 and 15) CV per patient were applied. To address imbalances in the dataset, data augmentation using adaptive synthetic (ADASYN) algorithm was implemented to ensure robust classification performance [21]. Data augmentation was specifically applied to the training set after the CV. The test set was not augmented. Evaluation metrics such as sensitivity, specificity, accuracy, and the AUC were computed to assess the effectiveness of the classifiers.

## Results

The processing of “prior” and “current” mammograms resulted in a reduction in image background, effectively removing areas that remained unchanged between screenings. The subtracted images demonstrated an average contrast ratio about two times higher than the “current” mammographic views with the same pre-processing (Fig. S2). It is important to note that none of the malignant masses was removed during this process (Fig. 5).

Feature selection, using a majority-rule-based combination of all ranking methods, identified a combination of 14 features which provided the best classification results (Table 2). Nine classifiers were employed for the classification of the segmented ROIs as normal or possible “future” malignancies. For *k*-nearest neighbors (*k*-NN), the number of nearest neighbors tested were 1, 3, 5, 7, 9, and 11. For SVMs, linear, polynomial, and radial basis function (rbf) kernels were assessed. In ensemble voting, both hard and soft voting schemes were evaluated, incorporating the classifiers with the highest performance. Voting combined linear discriminant analysis (LDA), *k*-NN (*k* = 9), SVMs (polynomial kernel), naïve bayes (NB), and extra trees (ET), in a soft-voting scheme. Furthermore, several artificial neural network (ANN) architectures were tested, with network parameters fine-tuned based on validation loss and accuracy. The selected ANN architecture consisted of a single hidden layer. The activation functions used were rectified linear unit (ReLU) for the input and hidden layers, and a softmax function for the output layer. The network was optimized using the Adam optimizer, with a batch size of 128 and a learning rate of 0.0001. The training process was conducted over 100 epochs.

**Fig. 6:**
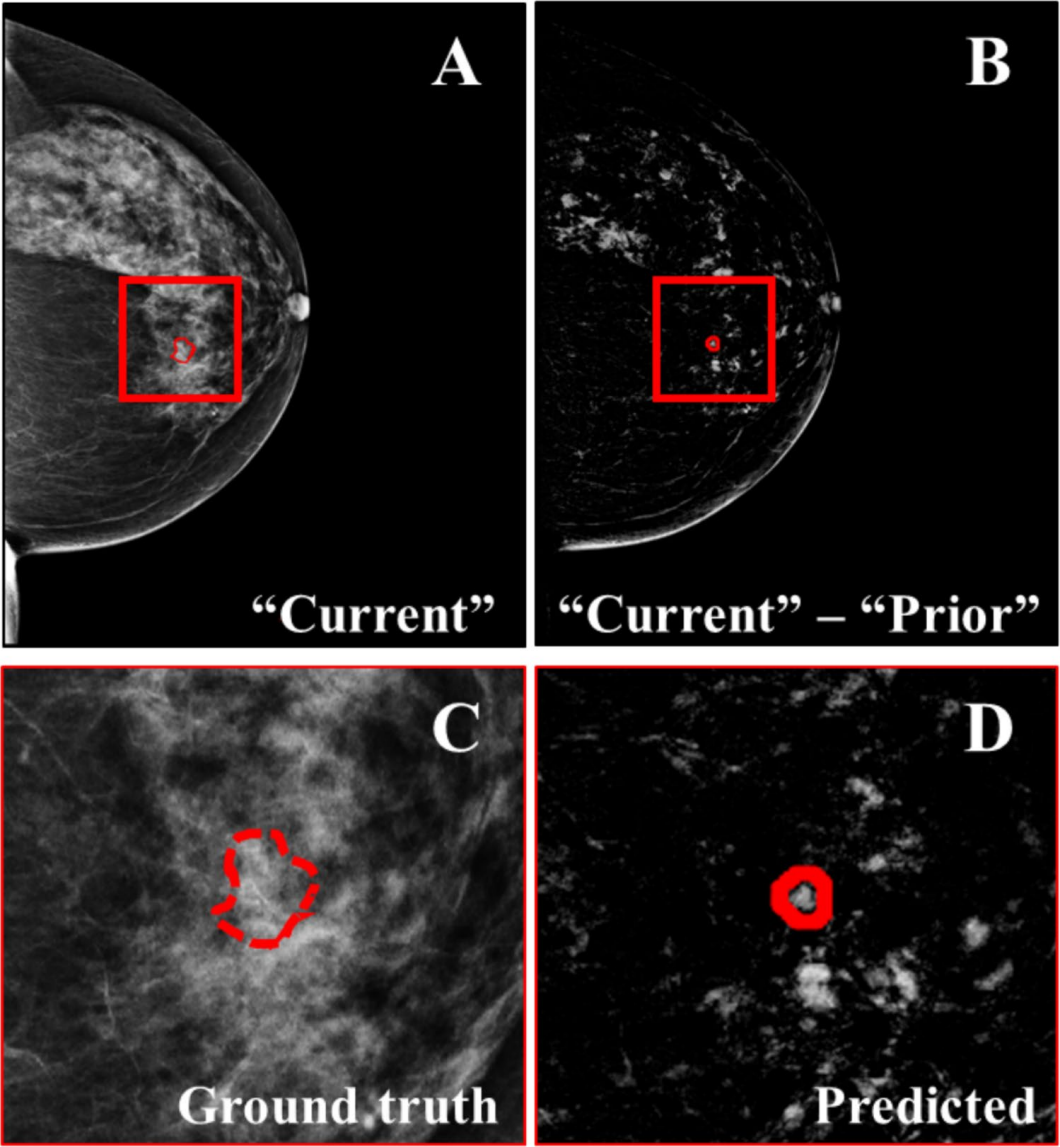
**A** “Current” mammogram showing the location where a mass would appear during the following screening round, found using image registration between the “current” and “future” images. There are no visible signs of malignancy there. **B** Difference image, after the subtraction of “prior” from “current,” showing the predicted “future” malignancy. **C** Zoomed region marked by the red square in **A**. **D** Zoomed region marked by the red square in **B**. No mass is radiologically visible in **A**, **B**, **C**, or **D**

The sensitivity, specificity, accuracy, and AUC in LOPO CV ranged between 82.3–93.4%, 96–99.8%, 93.8–99.6%, and 0.90–0.96, respectively (Table 3). For *k*-NN, *k* was set to 9 with a nearest tie-breaking algorithm. Figure 6 shows an example prediction of a “future” malignancy. The most successful classification scheme was based on ensemble voting, resulting in 93.6% (58/62) sensitivity, 98.8% (7179/7263) specificity, 98.8% (7237/7325) accuracy, and an AUC of 0.96. The classifiers were ranked first based on sensitivity, which is crucial for correctly identifying patients with BC. Then, the remaining metrices were considered (i.e., accuracy, specificity, and AUC). Statistical analysis was conducted to determine the significance of the observed performance differences among the various classifiers. Using the extended McNemar test [22], the performance of ensemble voting emerged as statistically significant when compared to all classifiers (*p* < 0.001), except *k*-NN and multi-layer perceptron (MLP) (*p* > 0.05). In future studies, with a larger population, statistical analysis will be performed, in order to select the most appropriate classifier for clinical use.

**Table 3:** Comparison of the classification results for different classifiers using leave-one-patient-out cross-validation

Classifier	Sensitivity [%]		Specificity [%]		Accuracy [%]		AUC
Random forest	50/62	80.65%	7247/7263	99.78%	7297/7325	99.62%	0.90
Gradient boosting	51/62	82.26%	7227/7263	99.50%	7278/7325	99.36%	0.91
Multi-layer perceptron	53/62	85.48%	7177/7263	98.82%	7230/7325	98.70%	0.92
k-nearest neighbors (*k* = 9)	56/62	90.32%	7174/7263	98.77%	7230/7325	98.70%	0.95
AdaBoost	56/62	90.32%	7233/7263	99.59%	7289/7325	99.51%	0.95
Support vector machine	57/62	91.94%	6813/7263	93.80%	6870/7325	93.79%	0.93
Naïve Bayes	57/62	91.94%	7052/7263	97.09%	7109/7325	97.05%	0.94
Artificial neural network	57/62	91.94%	7221/7263	99.42%	7278/7325	99.36%	0.96
Linear discriminant analysis	58/62	93.55%	6973/7263	96.01%	7031/7325	95.99%	0.94
Ensemble voting	**58/62**	**93.55%**	**7179/7263**	**98.84%**	**7237/7325**	**98.80%**	**0.96**

The classification performance was also assessed through k-fold CV, employing both 5- and 15-fold. The number of folds was selected based on the number of patients, which was 75, to create an even number of folds per patient and to evaluate two different k-fold scenarios. The algorithm demonstrated consistent and robust performance for both schemes (Fig. S3). In 5-fold CV, ensemble voting reached 93.4% (58/62) sensitivity and 98.6% (7164/7325) accuracy, using a combination of quadratic discriminant analysis (QDA), *k*-NN (*k* = 7), SVMs (polynomial kernel), NB, and ET, in a hard-voting scheme. In 15-fold CV, ensemble voting reached 95.2% (59/62) sensitivity and 97.8% (7107/7325) accuracy using a combination of LDA, QDA, *k*-NN (*k* = 9), SVMs (polynomial kernel), and NB, in a soft-voting scheme.

## Discussion

This study demonstrated an automated algorithm to predict the emergence of a breast mass during the next mammographic screening round, leveraging the combination of temporally sequential mammogram subtraction and feature-based machine learning. Through an analysis spanning between 2020 and 2024, data from 75 cases, each consisting of three consecutive digital mammograms, were collected from women aged 46 to 79. The ground truth was established by registering the “future” and “current” mammograms to precisely identify the location of the “future” malignant mass in the “current” image. The implementation of Demons registration proved highly effective in aligning sequential mammographic views, successfully compensating for inherent transformations and distortions occurring between screenings. This approach led to an improvement of approximately two times in contrast ratio, significantly enhancing the visibility of changes in the mammographic images. For the classification, the most accurate and robust classifier was ensemble voting, with 98.8% (7237/7325) accuracy, 93.6% (58/62) sensitivity, 98.8% (7179/7263) specificity, and 0.96 AUC. Notably, in 15-fold CV, the sensitivity increased, reaching 95.2% (59/62) and an average of 0.02 false positives per image. This performance indicates that the algorithm maintains high performance even when trained with fewer patients.

Despite having less than 100% accuracy, the proposed approach would likely have minimal consequences if applied clinically. Out of 62 malignancies, 4 were not predicted as such. However, this had an impact on only 2 out of 34 patients. Another two patients were correctly classified as at risk for “future” malignancy using another view (CC or MLO); thus, their care would not have been compromised. The emphasis on sensitivity underscores the importance of minimizing the number of patients incorrectly classified as disease-free. In k-fold CV, the overall performance was similar to LOPO with high classification accuracy. Although unlikely, a mass that has not changed between screenings could be subtracted and disappear from the final image. However, this would not impose serious clinical consequences since in cases of non-progressing lesions only a recall is recommended.

The utilization of subtraction of temporally sequential mammograms for the prediction of near-term BC occurrence is a newly developed approach, making direct comparisons with existing studies challenging. Variations in datasets, pre-processing methods, CV techniques, classifiers, and performance evaluation methodologies further complicate such comparisons. Previous studies have developed algorithms for near-term BC risk prediction using sequential mammograms, but each used different datasets, pre-processing methods, and classifiers [12, 13, 15–17, 23]. Directly comparing state-of-the-art algorithms is also difficult due to variations in validation methods and performance evaluations. Some studies randomly divided ROIs into training and test sets, which could include areas from the same patient in both sets [24, 25]. In this study, such bias was avoided by performing validation on a per-patient basis.

Table 4 compares different state-of-the-art algorithms for near-term BC prediction, illustrating that the proposed algorithm outperforms existing ones even those using deep learning models (0.96 vs. 0.93 in [23]). In order to further validate the proposed approach, a direct comparison was conducted with an image-only DL model, proposed by Yala et al. [15] that provided the most promising results in the literature. Using the same technique (ResNet18) on this dataset, a 0.61 AUC was achieved that is close to the expected results (i.e., 0.70 AUC), albeit lower as expected due to the significantly smaller dataset. In DL, large datasets are crucial to ensure proper generalization and prevent overfitting. Given the limited number of cases in this dataset, the DL model could not be adequately trained to achieve the same performance. The model was trained for 20 epochs, with a batch size of 256, a learning rate of 0.001, and 15-fold CV, per patient. Data augmentation techniques were also applied, including random translations, to enhance generalization. The proposed methodology outperformed the image-only DL model of Yala et al. [15] underscoring the added value and robustness of incorporating temporal subtraction and advanced feature selection techniques in the prediction process. Also, as shown in Table 4, the proposed methodology also outperformed the Hybrid DL Model of Yala et al. [16], in which image information and risk factors were combined. The impressive results of this study are a combination of (1) temporal subtraction, which enhances the visibility of subtle changes in the breast over time, providing important information for near-term BC prediction, (2) exhaustive feature selection that involved ranking features using eight different feature selection techniques, certifying that only the most relevant features were included, leading to improved model performance, (3) data augmentation, using ADASYN, which generated synthetic samples for the minority class, thus, enhancing the classification performance and model robustness, and addressing the imbalance between the two classes in the dataset, and (4) precise annotation of each individual abnormality both benign and malignant, instead of using vague bounding boxes to define the ROIs that include the abnormalities.

**Table 4:** Comparison of other algorithms for the prediction of breast cancer using sequential mammograms and feature-based machine learning

Reference	Nu. of patients	Classifier	Validation method	AUC
**Feature-based**				
Sun et al. (2015)	340	Support vector machine	tenfold CV (per patient)	0.73
Tan et al. (2016)	335	Support vector machine	leave-one-out CV (per patient)	0.73
** Proposed**	**75**	**Ensemble voting**	**leave-one-out CV (per patient)**	**0.96**
**Deep learning**				
Yala et al. (2019)	39,571	Convolutional Neural Network	71,689–8554–8751 (per patient)	0.7
He et al. (2019)	5147	Deep Neural Network	tenfold CV (per patient)	0.93
Yala et al. (2021)	91,520	Convolutional Neural Network	210,819–25644-58,539 (per patient)	0.80
Yala et al. (2021)	125,991	Convolutional Neural Network	210,819–25644-128,793 (per patient)	0.79
Lee et al., (2023)	16,113	Convolutional Neural Network	8113–800-600 (per patient)	0.77

*CV* cross-validation

A significant limitation of this study is the relatively small and unbalanced dataset. Data collection is challenging since three consecutive rounds of mammograms are needed for each participant, requiring four or more years of follow-up without interruptions. In addition, if a mass is detected in the last screening, the two previous screenings must be normal. For cases with suspicious masses, biopsy confirmations are also necessary to verify the malignancies. Similarly, for cases with benign abnormalities, follow-up mammography is needed to confirm the benign nature of the mass in the next screening round. Furthermore, another key limitation of this study is the lack of external validation using publicly available datasets. Unfortunately, publicly available databases are not suitable as they do not include three rounds of mammograms per patient, precisely detailed annotations for benign and malignant abnormalities, biopsy confirmations, and in some cases, they include scanned, low-resolution images. Another limitation is potential disagreements regarding the extent of mass areas as more experts annotate the same mammograms. Since the purpose of this study is to identify the appearance of “future” malignancies and not precisely annotate their extend, this would have minimal effect on the performance of the algorithm, but will be verified in future studies.

The promising results of this study warrant further investigation with a larger dataset that will include a broader age range and more representative samples. Additional prior exams can also be incorporated into the analysis, to extend the timeframe of evaluation and prediction. In the future, the benefit of additional normal prior rounds on the prediction of near-term BC occurrence will be evaluated.

Moreover, since most hospitals are now embracing digital breast tomosynthesis (DBT) along with mammography for patients with suspicious findings, the proposed technique should be extended to DBT. Furthermore, with a larger dataset, deep learning should be evaluated using both mammograms and DBT.

## Conclusion

This methodology holds the potential to automatically predict the appearance of breast abnormalities in the subsequent mammographic screening, even in the absence of visible lesions in the current mammogram. The clinical application of this algorithm has the potential to significantly improve the management of BC, with increased vigilance, ultimately leading to earlier detection, timely intervention, and improved patient outcomes. Its incorporation into clinical practice could pave the way for better prognosis and lower mortality rates.

## Supplementary Information

Below is the link to the electronic supplementary material.

Supplementary file1 (DOCX 863 KB)

## Data Availability

The datasets used and/or analyzed during the current study are available from the corresponding author on reasonable request.

## Abbreviations

BC: Breast cancer
LOPO: Leave-one-patient-out
CV: Cross-validation
