# Supplementary material for: Subtraction of Temporally Sequential Digital Mammograms: Prediction and Localization of Near-Term Breast Cancer Occurrence
Source: J Imaging Inform Med. 2025 Mar 7;38(6):4243–54. doi: 10.1007/s10278-025-01456-z (PMC12701212; doi:10.1007/s10278-025-01456-z)
Supplement: Supplementary file 1 — Supplementary file1 (DOCX 863 KB) [file 10278_2025_1456_MOESM1_ESM.docx]

# Supplementary Material

| 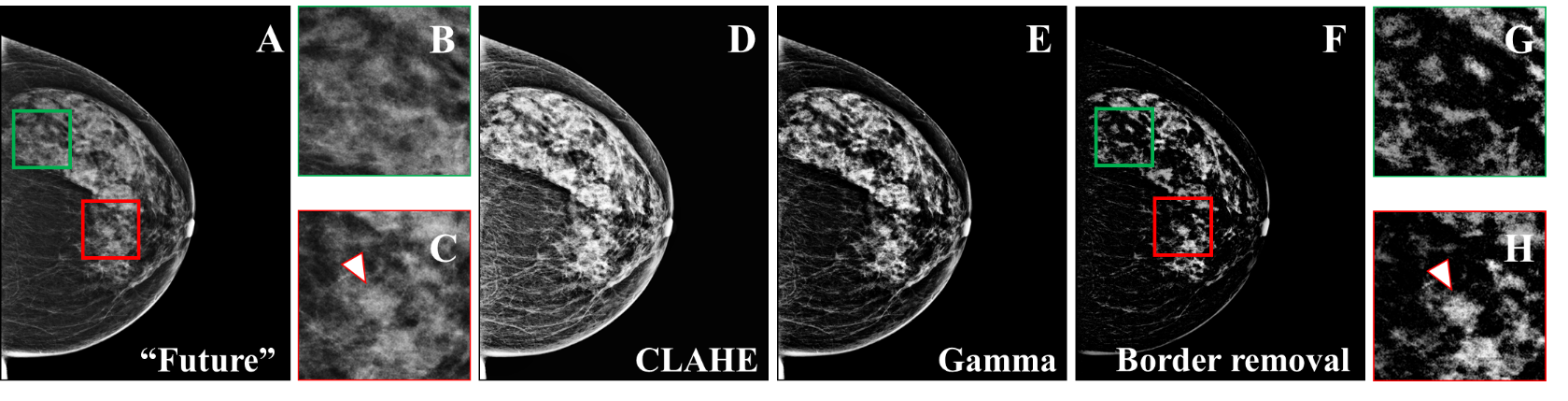 |
| --- |
| **Fig. S1** Effect of the pre-processing (BI-RADS breast density category *c*). **(A)** Original most recent mammographic view (the so-called “future” mammogram). **(B)**  Zoomed region marked by the green square in **A,** showing an area without masses. **(C)** Zoomed region marked by the red square in **A,** showing an area with a malignant mass (indicated by the arrow). **(D)** Image after contrast limited adaptive histogram equalization (CLAHE). **(E)** Image after gamma correction. **(F)** Final pre-processed image after border removal. **(G)**  Zoomed region marked by the green square in **F**, showing the same area as **B**, after pre-processing. **(H)**  Zoomed region marked by the red square in **F**, showing the same area as **C**, after pre-processing. *CLAHE:* contrast limited adaptive histogram equalization |

| 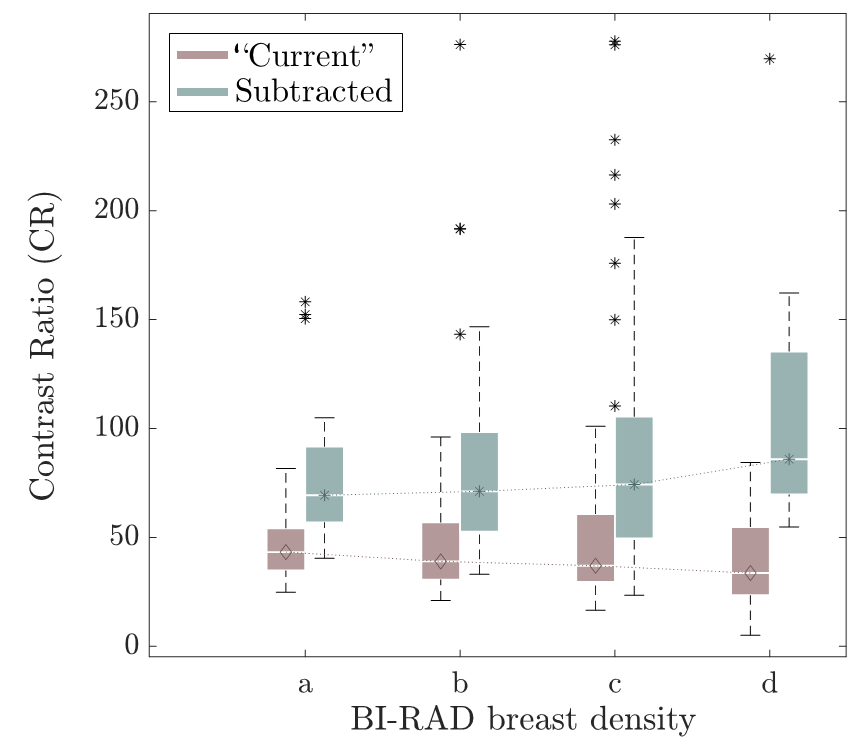 |
| --- |
| **Fig. S2** Box plot comparing the contrast ratio (CR) between subtracted images and the “current” images after pre-processing, for the four categories of breast density as defined by BI-RADS. |

| 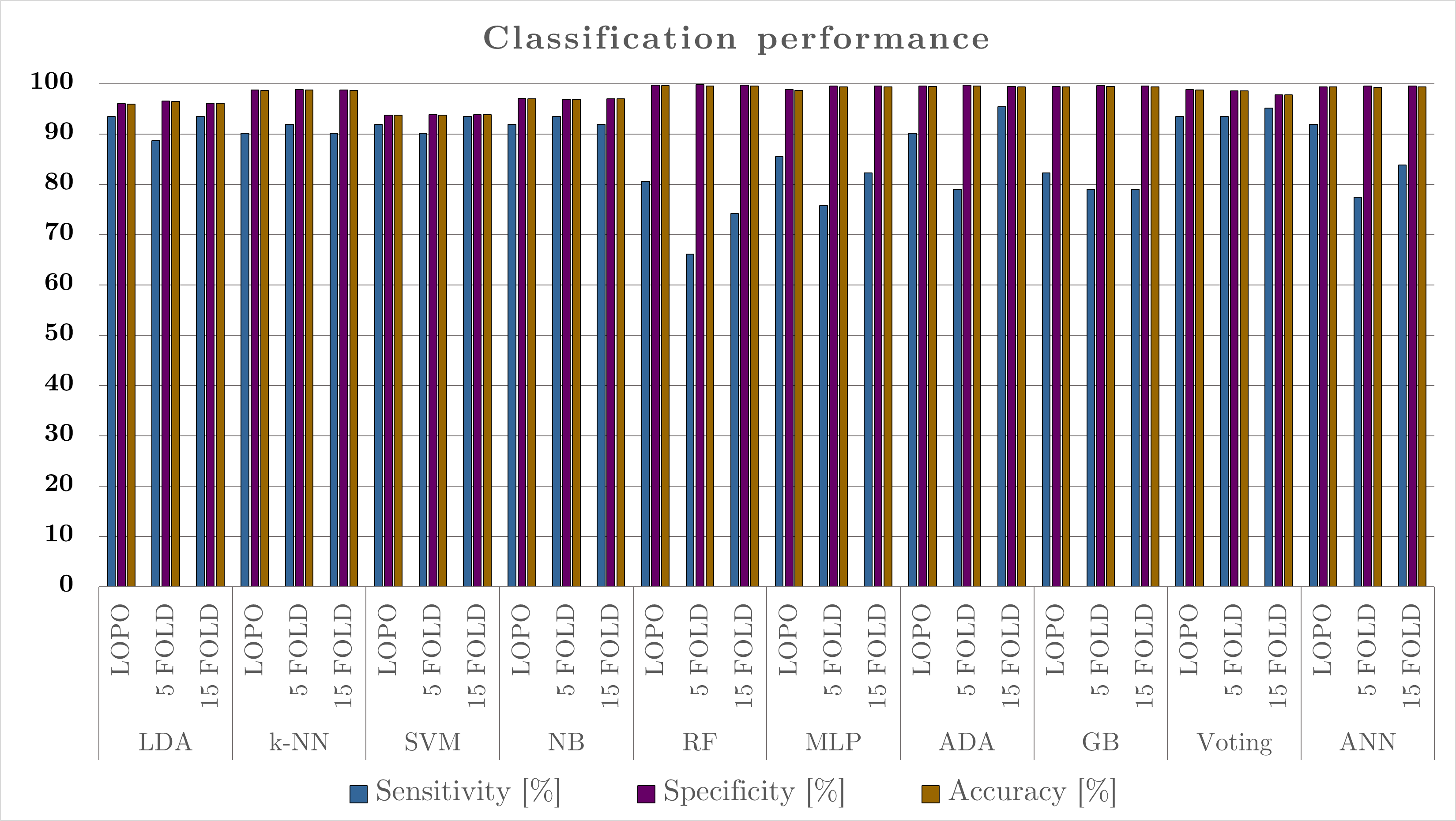 |
| --- |
| **Fig. S3** Classification results using different classifiers and cross-validation methods. *LDA:* linear discriminant analysis; *k-NN:* k-nearest neighbors; *SVM:* support vector machines; *NB:* naïve bayes; *RF:* random forest; *MLP:* multi-layer perceptron; *ADA:* adaboost; *GB:* gradient boosting; *ANN:* artificial neural network; *LOPO:* leave-one-patient-out |
